# Combining generative modelling and semi-supervised domain adaptation for whole heart cardiovascular magnetic resonance angiography segmentation

**DOI:** 10.1186/s12968-023-00981-6

**Published:** 2023-12-20

**Authors:** Marica Muffoletto, Hao Xu, Karl P. Kunze, Radhouene Neji, René Botnar, Claudia Prieto, Daniel Rückert, Alistair A. Young

**Affiliations:** 1 School of Biomedical Engineering and Imaging Sciences, King’s College, St Thomas’ Hospital, 4th Floor Lambeth Wing, Westminster Bridge, London, SW1 7EH UK; 2 MR Research Collaborations, Siemens Healthcare Limited, Frimley, UK; 3 Biomedical Image Analysis Group, Department of Computing, Imperial College London, London, UK; 4 Institute for Artificial Intelligence and Informatics in Medicine, Klinikum Rechts der Isar, Technical University of Munich, Munich, Germany

**Keywords:** Deep learning, Whole-heart segmentation, Domain adaptation, Generative adversarial networks, Variational auto-encoders

## Abstract

**Background:**

Quantification of three-dimensional (3D) cardiac anatomy is important for the evaluation of cardiovascular diseases. Changes in anatomy are indicative of remodeling processes as the heart tissue adapts to disease. Although robust segmentation methods exist for computed tomography angiography (CTA), few methods exist for whole-heart cardiovascular magnetic resonance angiograms (CMRA) which are more challenging due to variable contrast, lower signal to noise ratio and a limited amount of labeled data.

**Methods:**

Two state-of-the-art unsupervised generative deep learning domain adaptation architectures, generative adversarial networks and variational auto-encoders, were applied to 3D whole heart segmentation of both conventional (n = 20) and high-resolution (n = 45) CMRA (target) images, given segmented CTA (source) images for training. An additional supervised loss function was implemented to improve performance given 10%, 20% and 30% segmented CMRA cases. A fully supervised nn-UNet trained on the given CMRA segmentations was used as the benchmark.

**Results:**

The addition of a small number of segmented CMRA training cases substantially improved performance in both generative architectures in both standard and high-resolution datasets. Compared with the nn-UNet benchmark, the generative methods showed substantially better performance in the case of limited labelled cases. On the standard CMRA dataset, an average 12% (adversarial method) and 10% (variational method) improvement in Dice score was obtained.

**Conclusions:**

Unsupervised domain-adaptation methods for CMRA segmentation can be boosted by the addition of a small number of supervised target training cases. When only few labelled cases are available, semi-supervised generative modelling is superior to supervised methods.

**Supplementary Information:**

The online version contains supplementary material available at 10.1186/s12968-023-00981-6.

## Introduction

Accurate quantification of whole heart anatomy is required for patient diagnosis and prognosis as well as the evaluation of treatment. Non-invasive medical imaging techniques such as computed tomography angiography (CTA) or cardiovascular magnetic resonance angiography (CMRA) can be used to quantify 3D heart anatomy [16, 26]. Deep Learning techniques can give highly accurate segmentations given sufficient ground truth labels [2, 6, 25] even in the presence of low contrast and high noise images [12, 15]. Despite rapid advancements, a major unsolved problem is the poor adaptability of these methods to different imaging modalities, scanners and acquisition protocols, and the related need for large amounts of labelled data in each domain. Deep learning models trained on one domain do not generalize well to a different target domain [1] with zero or very few labelled cases. The difference in distribution between a large, labelled source domain and an unlabeled target domain is called “domain gap" [10, 22].

Here, we investigate how to bridge the domain gap between CTA images used as source and CMRA images as target. Given sufficient manual domain-specific ground truth labelled data, the UNet architecture had provided state-of-the-art performance in whole heart segmentation applications [25]. In particular, the nn-UNet package provides a self-configuring solution with a range of data augmentation and ensembling tools [11]. However, these models typically do not generalize to other domains. Unsupervised domain adaptation (UDA) methods seek to transfer segmentation ability acquired in one domain to the other without the need for ground truth labels in the target domain. Most UDA methods use generative modelling, in which the domain gap is reduced by generating target domain images from source domain images. Two powerful UDA generative modelling methods are variational autoencoders (VAEs) and generative adversarial networks (GANs). VAEs use probabilistic encoder and decoder networks, optimized using maximum likelihood. Conversely, GANs use generator and discriminator networks which are optimized in an adversarial manner. It is currently unclear which architecture is best for cardiac CMRA applications. Two methods which have provided state-of-the-art performance in whole heart segmentation UDA applications are the synergistic image and feature alignment (SIFA) GAN architecture [7, 8] and the variational approximation for domain adaptation (VARDA) VAE architecture [20] which have both shown greater success in CMRA (source) to CTA (target) domain transfer than vice-versa.

In this paper we improve our preliminary work [14] investigating semi-supervised performance of these two methods, by removing the requirement for pre-processing registration between CTA and CMRA datasets, incorporating a fivefold cross validation for a full statistical analysis, testing a range of supervision levels from 0 to 30%, testing performance on two different CMRA protocols, and comparing with the supervised nn-UNet method. This study is designed to be applied to multi-domain data with a variable number of supervised cases, and to provide a guideline for choosing the best approach for this challenging problem.

## Materials and methods

### Data acquisition

**Fig. 1:**
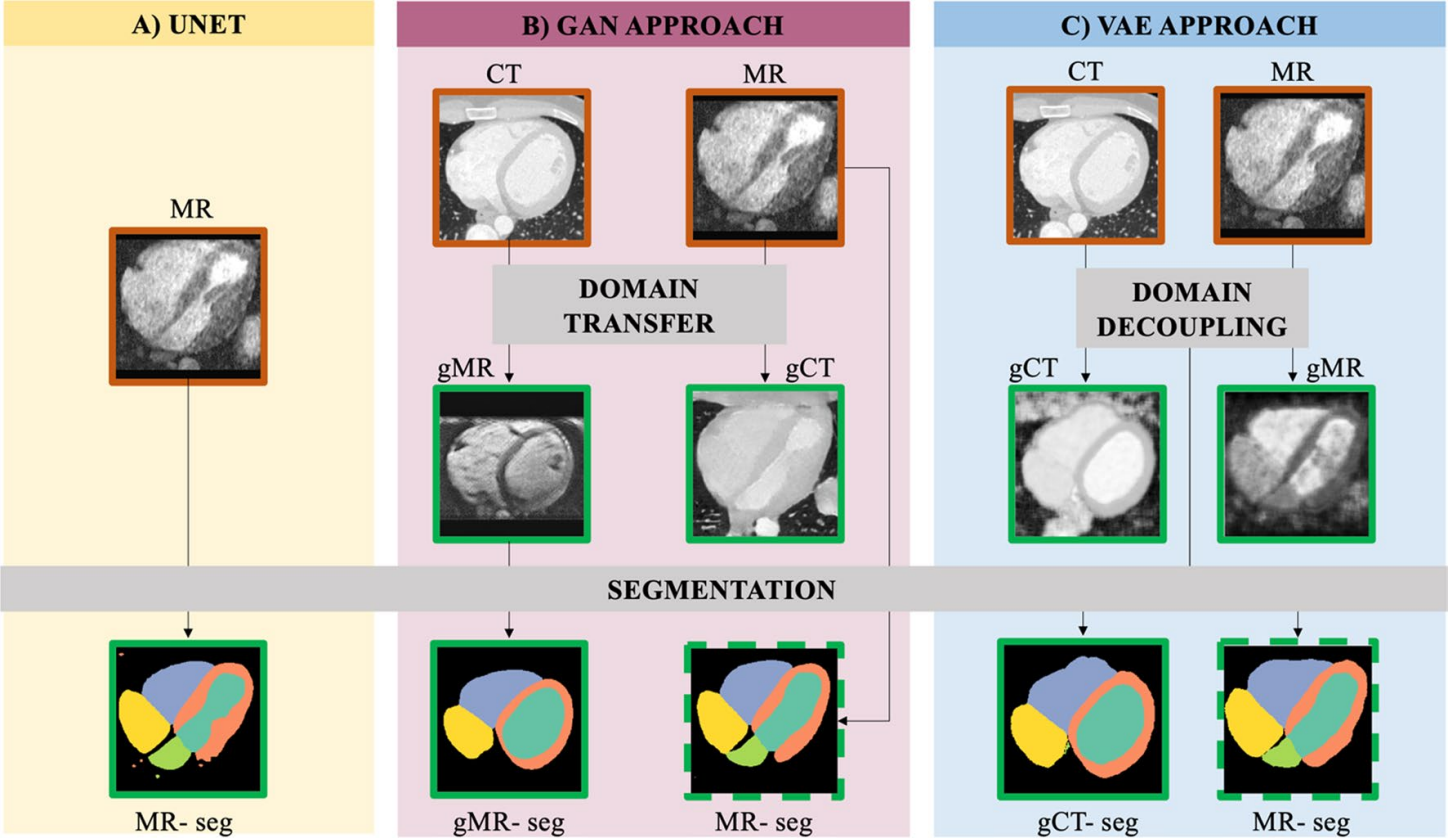
Summary of options to obtain an automatic CMRA segmentation. The border in each image is orange for inputs to the deep learning networks, and green for outputs. Dashed borders indicate that the outputs can be generated with or without corresponding ground truth label maps. Option **A** is a fully supervised approach (UNet). It only relies on a segmentation module, which takes as input MR images and corresponding label maps to produce as output the segmentations MR-seg. Option **B** and **C** are generative modelling approaches; **B** is a GAN approach which uses the Domain Transfer module plus a Segmentation module. CT and MR images are used as inputs to generate gMR and gCT respectively. gMR and MR are fed into the segmentation module which learns how to segment images from this domain, whether they are real or generated, utilizing the CT label maps. If available, MR label maps can also be used as a supervised segmentation loss, in any quantity; **C** is a VAE approach which uses the domain decoupling module to generate gCT and gMR images with no domain-specific features. As before, the Segmentation module can be trained with or without ground truth MR-seg, but it requires CT label maps. CMRA: cardiovascular magnetic resonance angiography; GAN: generative adversarial network; VAE: variational autoencoder

Figure 1 is a summary of the methods we investigate, where inputs and outputs are color-coded to show what is required and what is offered by each method. The first dataset comprised standard CMRA images from the multi-modality whole heart segmentation (MMWHS) cardiac segmentation challenge 2017 [13, 24, 25] which is publicly available. This includes 20 unpaired CMRAs and CTAs with ground truth labels. The cardiac CT data were acquired using routine CT angiography, covering the whole heart from the upper abdominal to the aortic arch. Slices are acquired in the axial view. The in-plane resolution was approx 0.78 × 0.78 mm and the average slice thickness was 1.60 mm. The CMRA data were acquired using 3D balanced steady state free precession (b-SSFP) sequences, without contrast, with approx. 2 mm acquisition resolution in each direction and reconstructed (resampled) into approx 1 mm [25]. Respiratory gating was performed using a navigator placed on the diaphragm and cardiac gating was performed retrospectively from the ECG.

The second dataset [4] included 51 paired cases of CTA and CMRA, with patients and healthy subjects. The acquisition of the 3D whole-heart isotropic sub-millimeter resolution CMRAs was performed using free-breathing bSSFP with image navigator (iNAV) and non-rigid motion compensated reconstruction, described in [4]. The CMRA images were reconstructed to $0.6\,{\text{mm}}^{3}$ isotropic resolution, while the CTAs had 0.5 mm slice thickness, and in-plane resolution of 0.2 $\sim$ 0.4 mm × 0.2 $\sim$ 0.4 mm. To obtain the labels for the CTA dataset, we used the method described in [21]. Ground truth segmentations for the CMRA cases were obtained by registering the CT with the MR using non-rigid registration [17] and manually correcting the resulting segmentation errors using 3D Slicer. Note that the registration was only used to generate ground truth CMRA label maps. Unlike our previous work [14], the current methods did not require paired CTA and CMRA datasets and did not perform registration as a pre-processing step. We will refer to this second dataset as High Resolution CMRA (HRMRA), to distinguish it from the MMWHS dataset, where the CMRA images have a lower resolution.

Labels of interest included all the following: ascending aorta (AA), left atrium blood cavity (LA), left ventricle blood cavity (LV), myocardium of the left ventricle (MYO), right ventricle (RV) and right atrium (RA).

### Network architectures and optimization

The SIFA architecture [7, 8] used a generator to perform a source-to-target image transformation, and a shared encoder which takes as inputs the real target ${x}^{t}$ or the generated target $\tilde{x}^{t}$ images, and it is connected to a decoder and a pixel-wise classifier. The former reconstructs both images into a generated source (similar to the CycleGAN architecture [23]), while the latter performs the image segmentation task. The model weights are optimised by a combination of adversarial losses, reconstruction losses and a source segmentation loss. More details are given in the Additional file 1.

The VARDA architecture [20] used two VAEs for encoding source and target domains. The total loss is a combination of two reconstruction terms, the Kullback–Leibler (KL) Divergence term, and a discrepancy loss which is introduced as an explicit metric to directly reduce differences between the latent variables from the two domains. The classifier takes features from the encoder and predicts a segmentation from both the source and target images. More details are given in the Additional file 1.

**Fig. 2:**
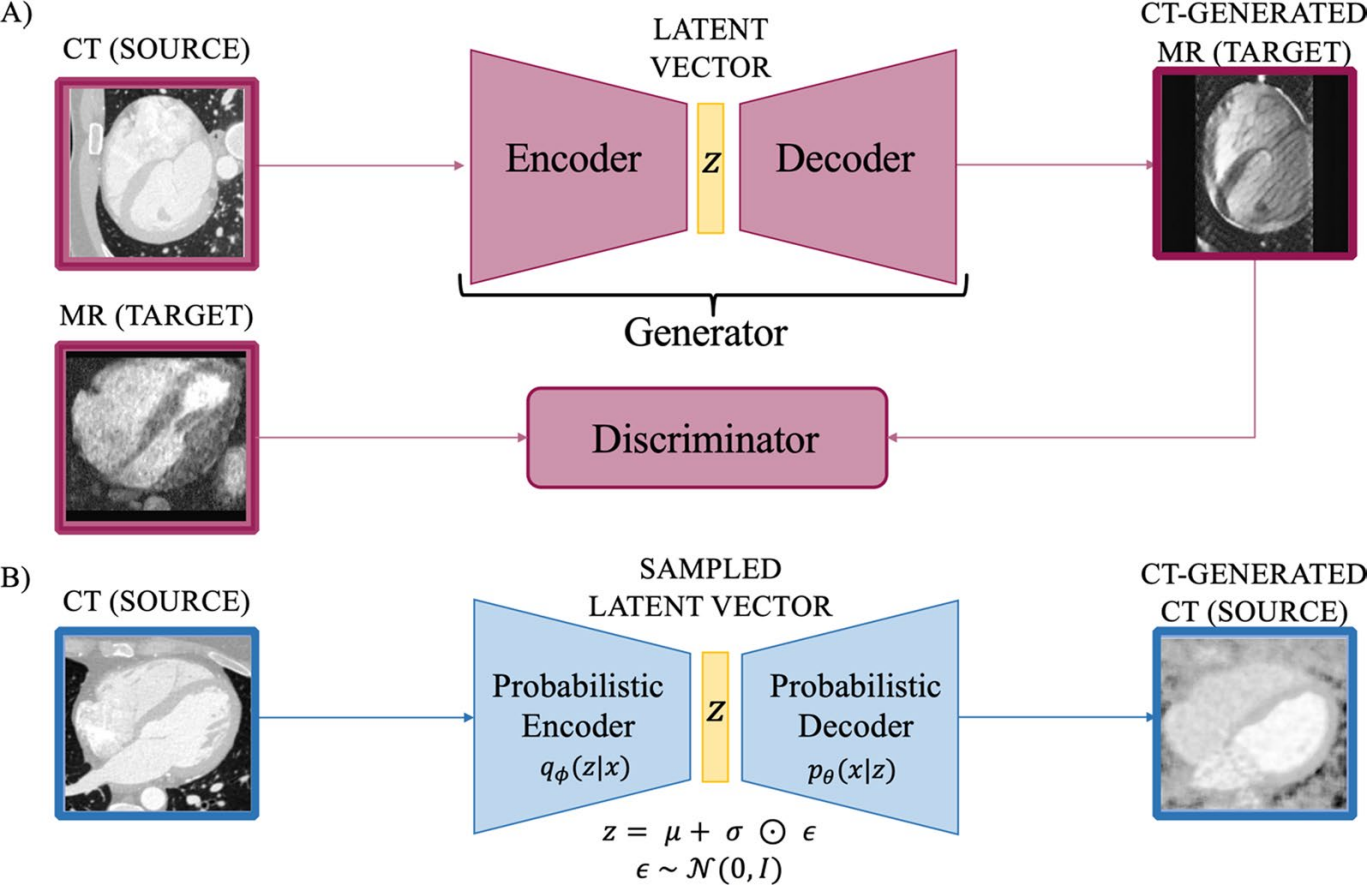
Basic diagrams of DL generative modelling architectures. **A** GAN structure where the Generator is decomposed into Encoder + Decoder. The Generator generates MR images from CT images, and the Discriminator discerns between real MR and CT-generated ones. **B** VAE structure which feeds the original input into a probabilistic encoder; the encoder learns vectors μ and σ from the data and the reparameterization trick is used to obtain a parametrised latent space z (note ⊙ is used for element-wise multiplication) from which images can be reconstructed. GAN: generative adversarial network; VAE: variational autoencoder

The input for both of these techniques was axial 2D slices from the 3D volumes in both datasets. Figure 2 shows the basic building blocks for the two techniques. Part A is a schematic illustration of a GAN applied to our scope. A generator model outputs an image whose quality is being optimized through the feedback given by a discriminator model. Its role is to differentiate between a real (true MRA) and fake image (CT-generated MRA). Part B represents a VAE, where the input is first fed into an encoder and then reconstructed through a decoder. The decoder samples from a latent vector drawn from a distribution with mean μ and standard deviation σ (Fig. 2). The generation of images is only possible through the regularization of the latent space using element-wise multiplication of the standard deviation σ with a random variable sampled from a Gaussian distribution N.

As the original methods described in [8, 20] are fully unsupervised, we modified both approaches to accommodate any number of supervised cases [14]. To do this, we introduced the following modifications to the original networks:

- in the GAN-based approach, where usually the segmentation loss relies on the source label map (since the transformation between source and target is learnt), we added true target labels. Hence, the segmentation loss was alternatively obtained by source-to-target label maps or real target label maps, depending on the input case;
- in the VAE-based approach, we used a similar technique. Here, the segmentation loss is originally just for the source image, we added one for the target, and we conditioned the reconstruction of the target image not solely on the predicted label map, but on the ground truth label map, when available.

The SIFA network was re-implemented in PyTorch, while VARDA was adapted from the PyTorch repository.

For comparison with a fully supervised network, we first ran the nnUNet package [11] on our tasks to obtain an optimization of processing steps and hyperparameters, then we used these to train our own implementation of a 2D Dynamic UNet, implemented with the medical open network for artificial intelligence (MONAI) framework [9]. To emulate the nnUNet training protocol, we added affine, Gaussian noise, Gaussian blur, scale intensity and mirror augmentation techniques provided by MONAI [9].

Prior to training, we resized and cropped each multi-modal image to focus only on the heart region, to obtain 256 × 256 image size for the GAN-based approach and 192 × 192 image size for the VAE-based approach. For the GAN-based approach we rescaled each axial slice to the range [− 1,1] as this substantially improves training of GANs since the generator activation layer is generally a tanh function which produces images in the range [− 1, 1]. For the VAE-based, we rescaled the data to have a mean of 0 and a standard deviation of 1 (standardization). Each slice was then fed to the networks in an unpaired fashion, hence there was no correspondence in anatomies between source and target domains.

### Evaluation setup

For each generative modelling approach (GAN-based and VAE-based), we performed 4 experiments: no supervision, 10%, 20%, and 30% supervision. In the MMWHS dataset this corresponded to 2, 4, 6 supervised target cases, while for the HRMRA we used 5, 10 and 15 cases respectively. Since UNet is a fully supervised method, we only run 3 experiments with 10%, 20%, and 30% supervision. Every label map obtained was compared to manual ground truth using 3D Dice and average surface distance (ASD) metrics. We used a fivefold cross validation for every experiment, with data splits performed by patient to avoid potential data leakage. Supervised cases for each fold were randomly picked excluding the validation cases. We compared all experiments to highlight the difference in levels of supervision or in approach (GAN, VAE, UNet) through the Wilcoxon paired test and corresponding p-values. For visualization, we used the Python stats toolkit from [5].

## Results

All experiments were evaluated through 3D Dice and ASD (mm) metrics. The results were aggregated over all test folds (Additional file 1: Tables S1, S2).

**Fig. 3:**
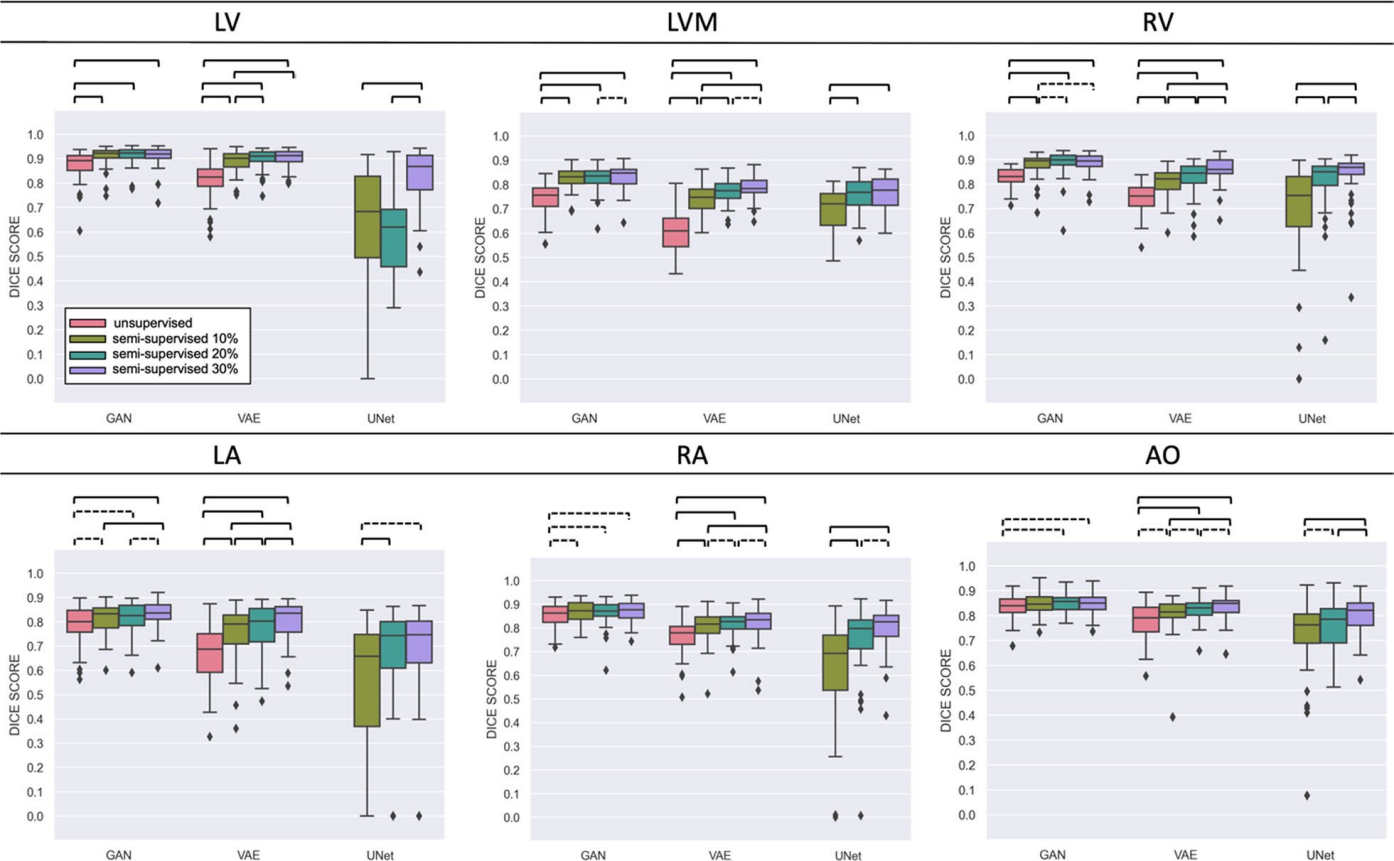
Results grouped by label. In each boxplot, statistical analysis is conducted between experiments with varying levels of supervision, as per legend on the top left corner. Dashed brackets for p <  = 5.00e−02, square brackets for p <  = 1.00e−03. HRMRA Dataset. HRMRA, high resolution magnetic resonance angiography

**Fig. 4:**
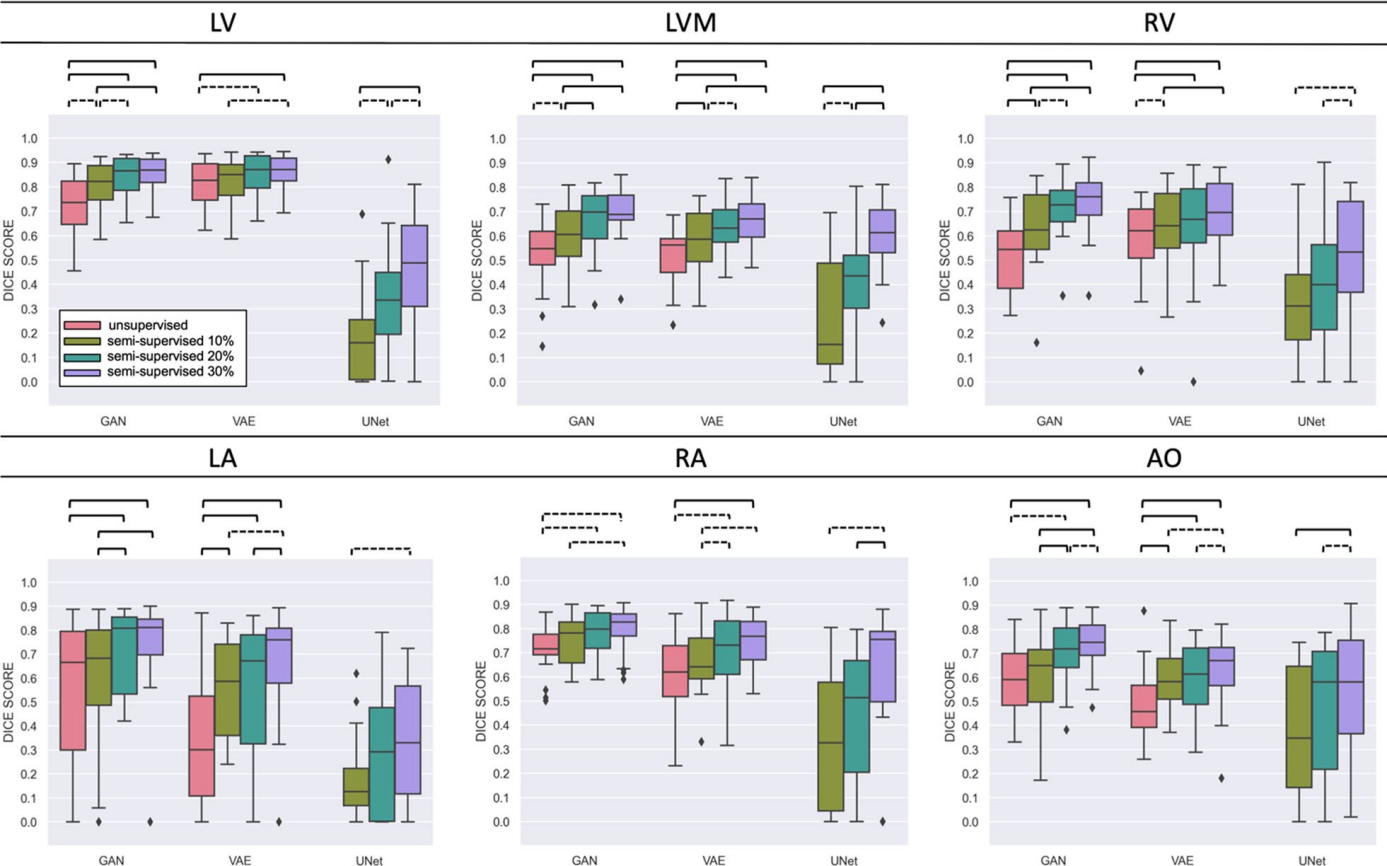
Results grouped by label. In each boxplot, statistical analysis is conducted between experiments with varying levels of supervision, as per legend on the top left corner. Dashed brackets for p <  = 5.00e−02, square brackets for p <  = 1.00e−03. MMWHS Dataset. MMWHS, multi-modality whole heart segmentation

We first provide an overview of the experiments in Figs. 3, 4. These show the effect of adding supervised target cases in the HRMRA and MMWHS datasets respectively. As expected, the performance of GAN, VAE and UNet were higher on the HRMRA dataset than on the MMWHS dataset. This is likely due to the availability of more cases for training, and the higher quality of the HRMRA images. ASD results showed similar patterns to Dice (Additional file 1: Tables S1 and S2). Comparing different levels of supervision per each label, the difference between no supervision and 30% supervision was almost always significant. This was true for all experiments, except for some labels in the UNet method, and for the RA/AO labels in the GAN-based results for the HRMRA dataset. A significant improvement was often found when going from no supervision to 10% supervision, especially in the HRMRA dataset, while it becomes rarer between 10% supervision and 20% supervision, and even more between 20 and 30% experiments.

Figure S1, S2 in Additional file 1 compare performance between methods. Both GAN and VAE significantly outperformed Unet for most labels and supervision levels. In the MMWHS dataset, GAN and VAE methods were not significantly different for LV, LVM and RV labels at 0%, 10%, 20% and 30% supervised target cases. However, GAN outperformed VAE for LA at 0% and 20%, RA at 0% and 10%, AO at 0%, 20% and 30% supervision ($p<0.05$). In the HRMRA dataset, GAN outperformed VAE for most labels at every level of supervision.

**Fig. 5:**
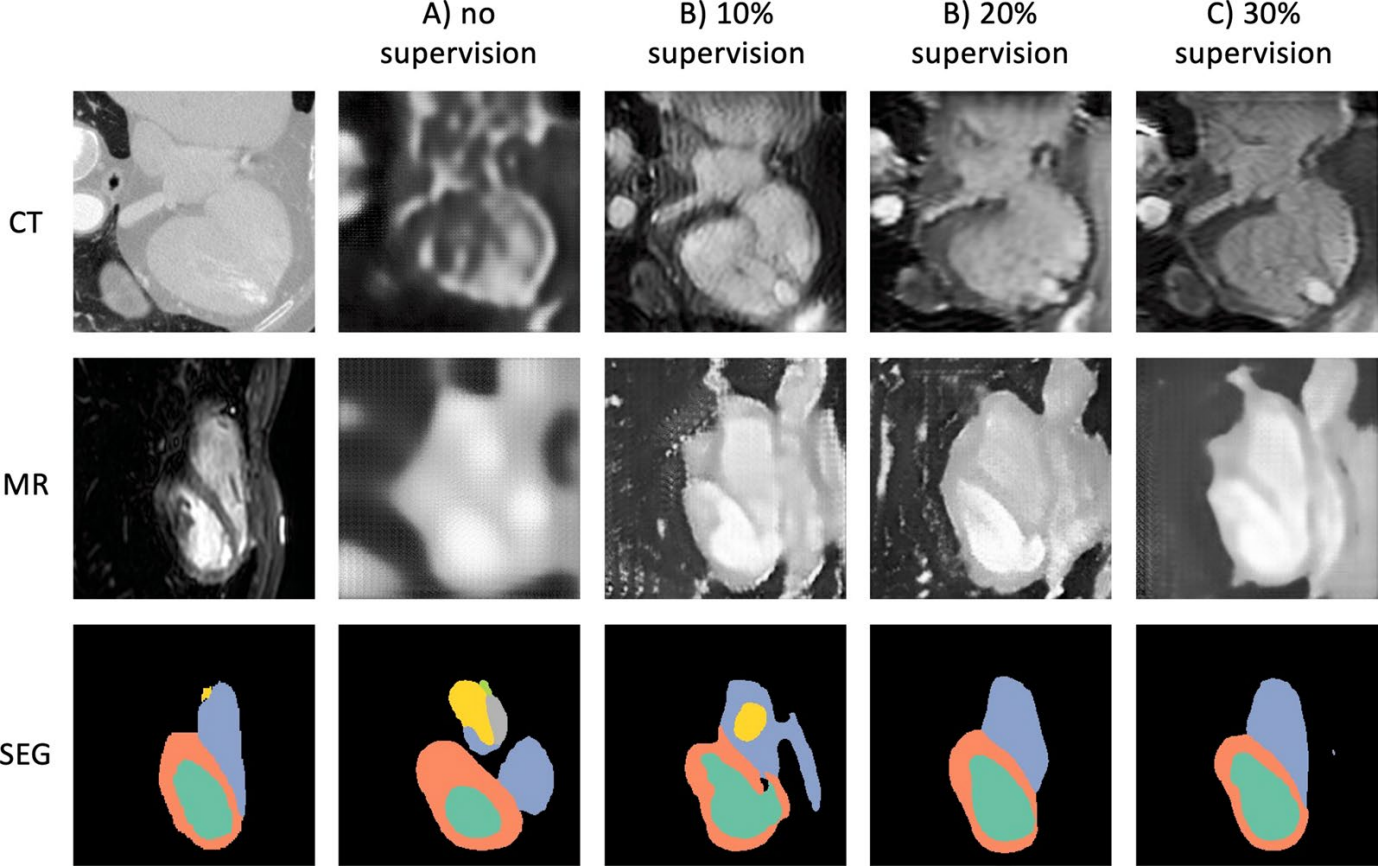
GAN-based method results on Dataset 1 (MMWHS). The first column shows the original input images, the following columns show outputs of the network for progressively higher level of supervision. Row 1–2 show generated images, while row 3 shows segmentation output. The GAN-based method transforms the source modality (CT) into target (MR) and vice versa. Legend for segmentation labels as follows: LV = turquoise, LVM = orange, RV = blue, LA = green, RA = yellow, AO = grey. GAN, generative adversarial network

**Fig. 6:**
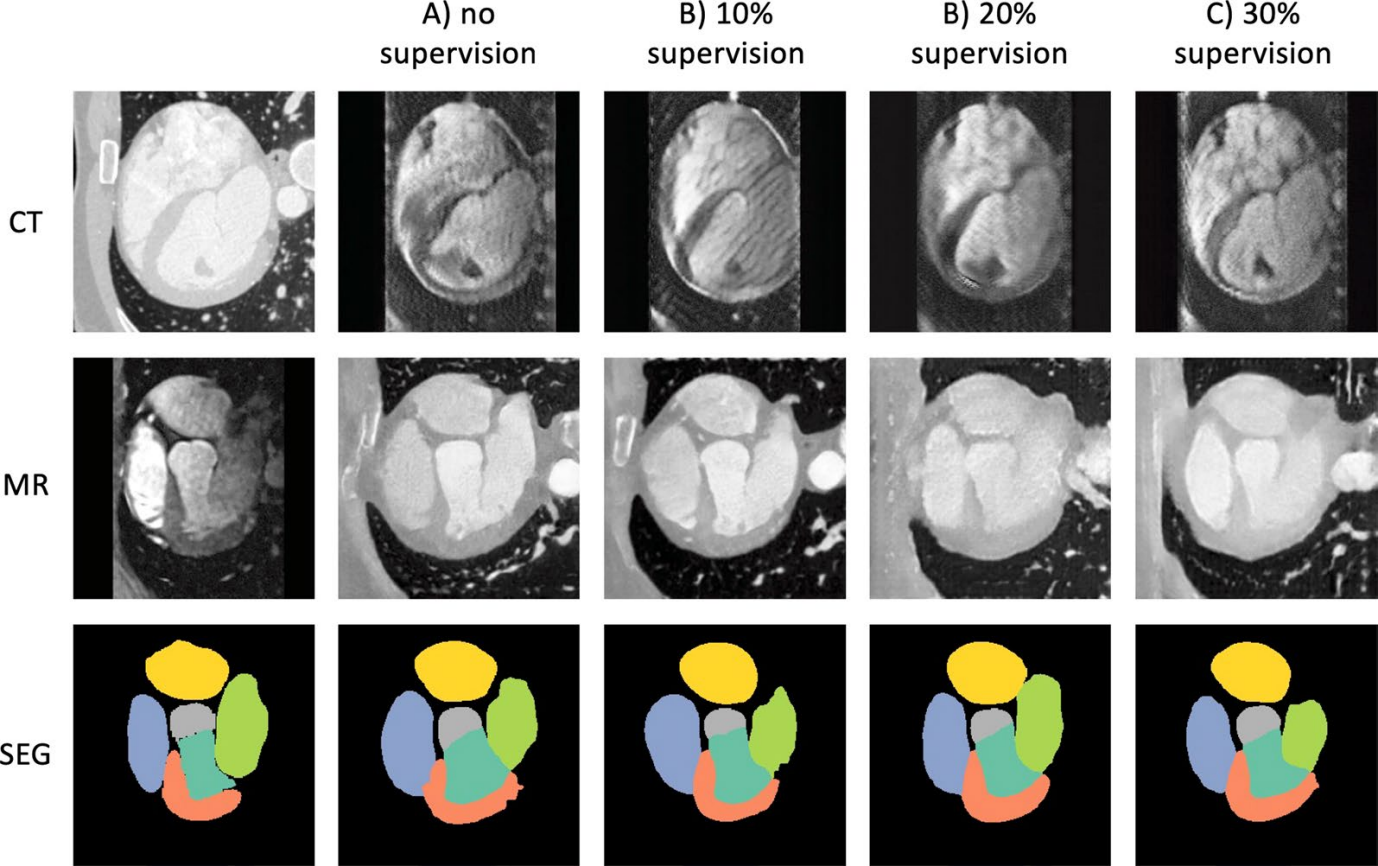
GAN-based method results on Dataset 2 (HRMRA). Explanation and legend for segmentation labels as above

**Fig. 7:**
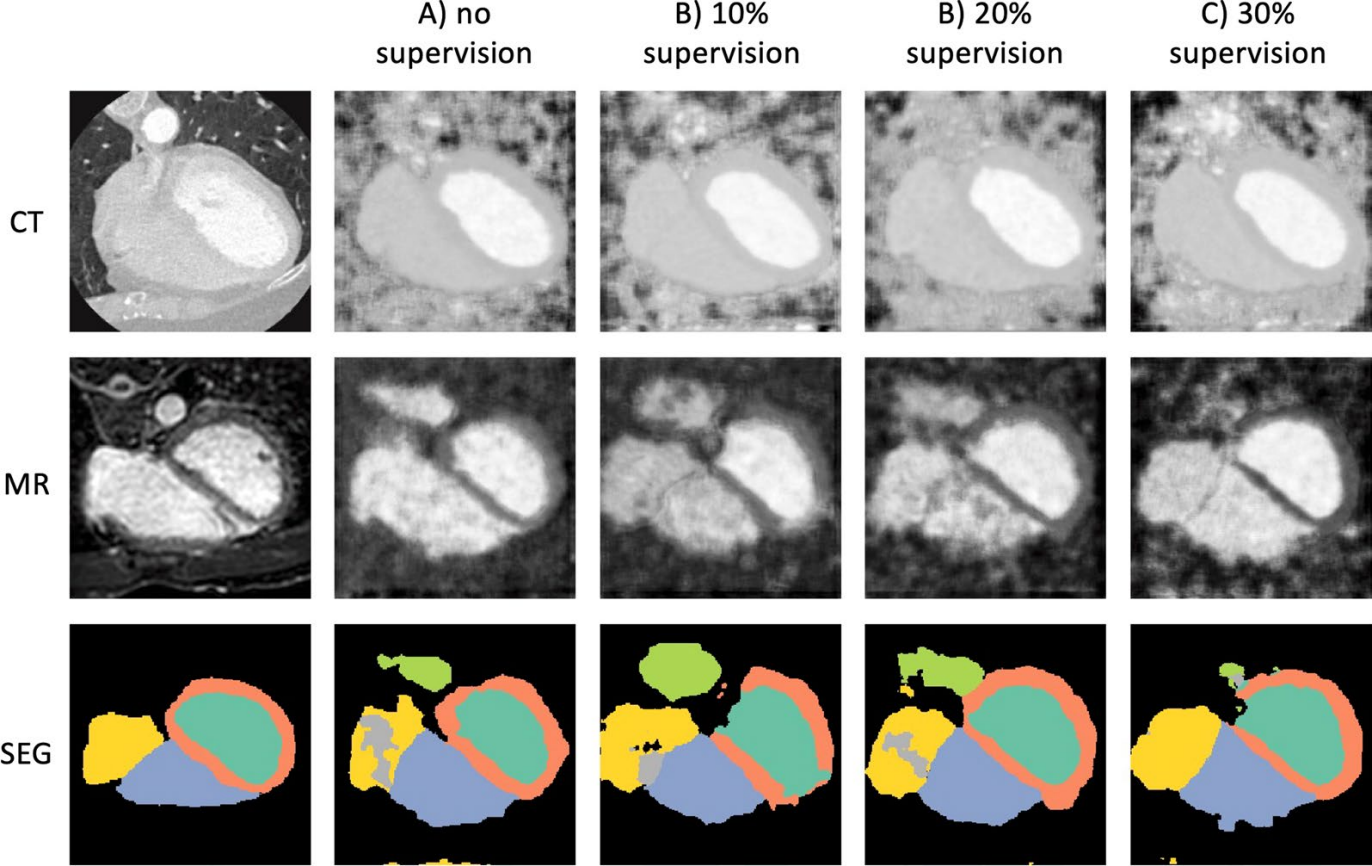
VAE-based method results on Dataset 1 (MMWHS). The first column shows the original input images, the following columns show outputs of the network for progressively higher level of supervision. Rows 1–2 show reconstructed images, while row 3 shows segmentation output. The VAE-based method tries to reconstruct a source (CT) and a target (MR) image which are modality-invariant. Legend for segmentation labels as follows: LV = turquoise, LVM = orange, RV = blue, LA = green, RA = yellow, AO = grey

**Fig. 8:**
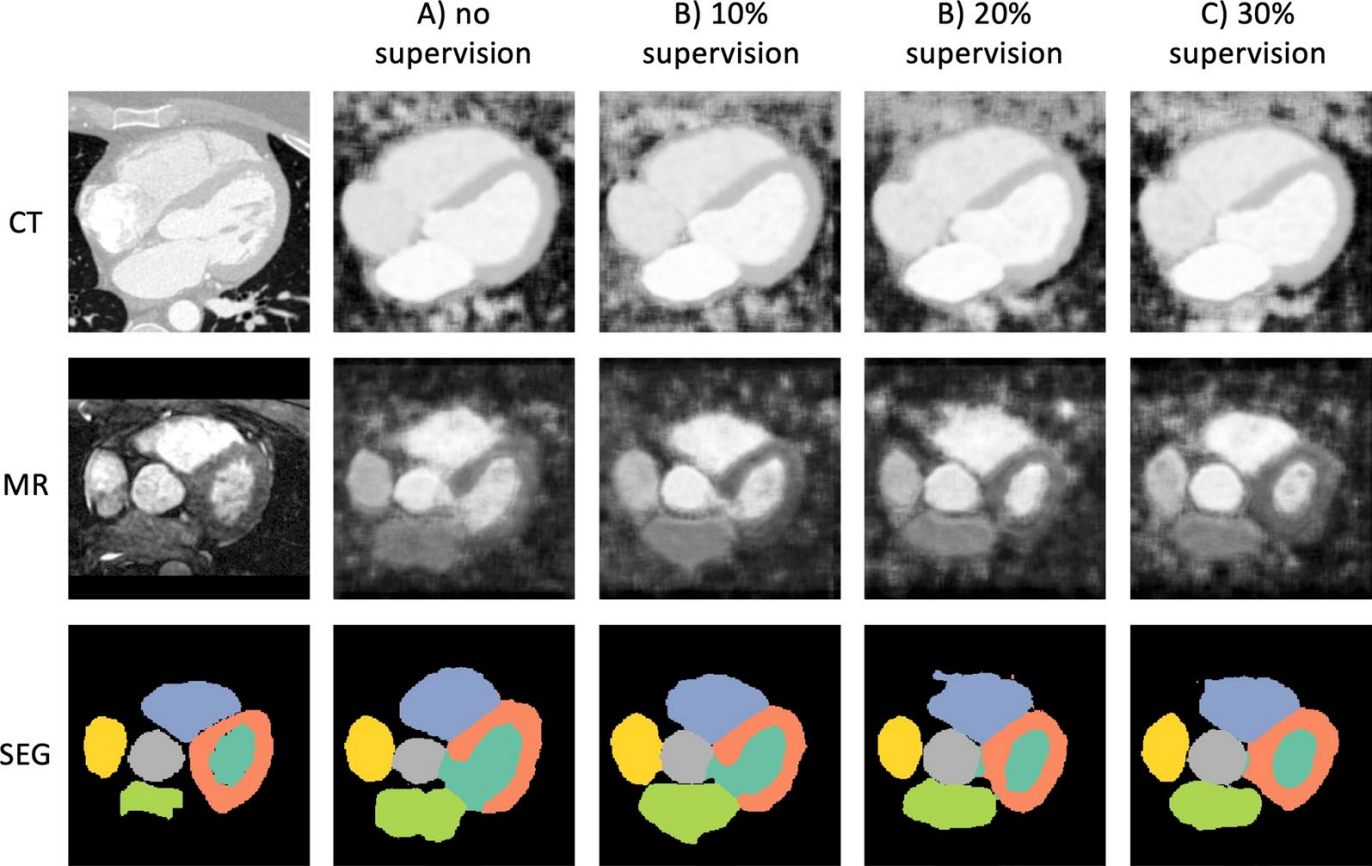
VAE-based method results on Dataset 2 (HRMRA). Explanation and legend for segmentation labels as above

Figures 5, 6, 7, 8 show examples of the outputs from the GAN-based method and the VAE-based method on each dataset. The first two rows show the images generated by each experiment for CTA or CMRA inputs, the last row shows the ground truth MR segmentation on the left and the predicted ones on the right. The difference in the two generative approaches can be observed. The GAN-based network tries to generate an CMRA from CTA, and vice versa, while the VAE generates less domain-dependent images due to the alignment of the joint latent space. From a comparison between Figs. 5 and 6, and between Figs. 7 and 8, it is also clearly visible that the MMWHS dataset represents a bigger challenge with greater impact of the supervision on the quality of the predicted labels. The difference is more subtle for the HRMRA dataset (Figs. 6 and 8), where less improvement in predicted segmentation quality is seen with increasing supervision, although still present (cf. Fig. 3 and Additional file 1: Table S2).

**Fig. 9:**
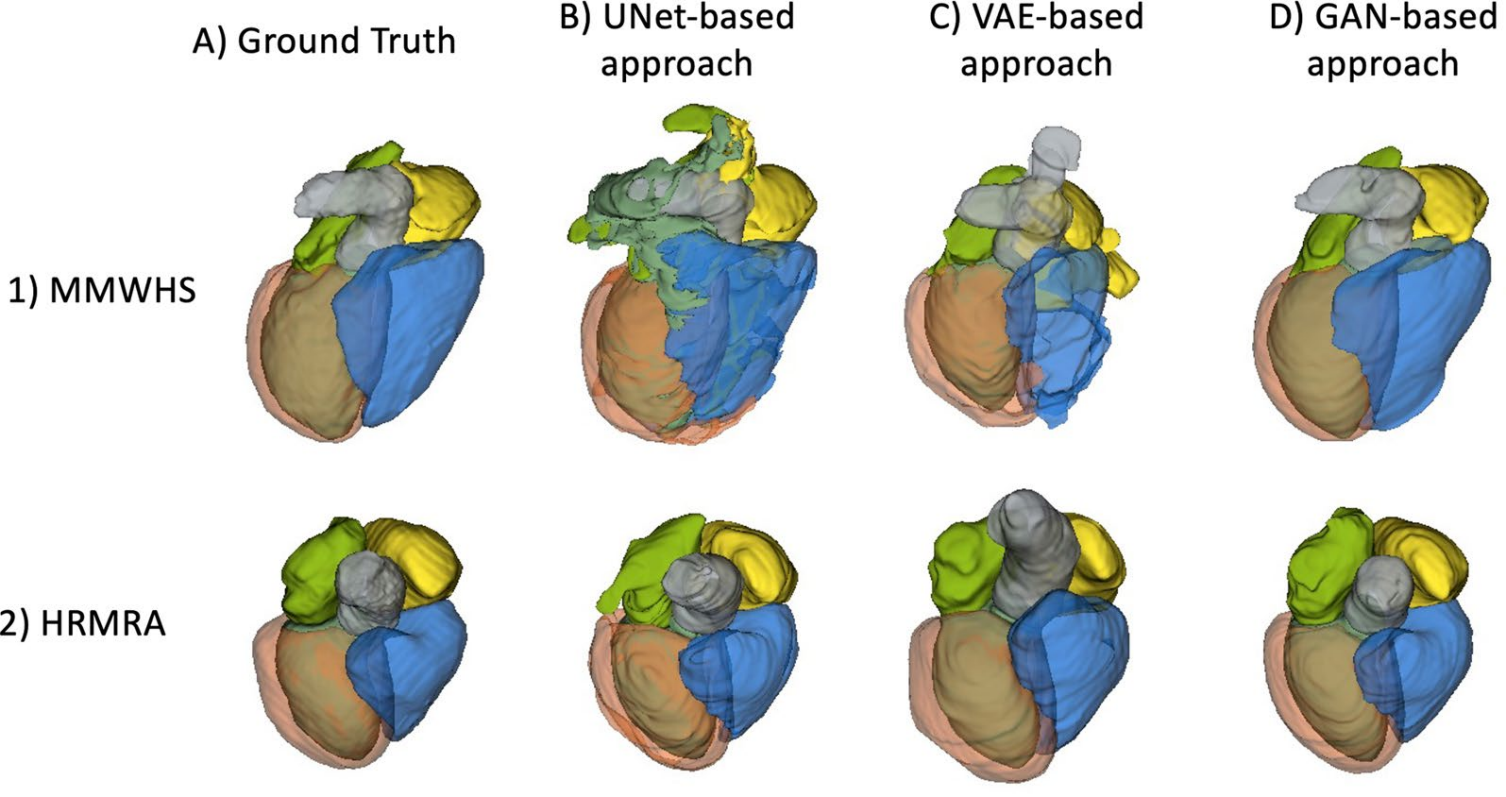
Comparison between ground truth 3D label maps and the results obtained using different approaches under a 30% supervision (maximum available). One random case was extracted from fold 1 in both dataset 1 and 2. Legend for segmentation labels as follows: LV = turquoise, LVM = orange, RV = blue, LA = green, RA = yellow, AO = grey

To validate the consistency across slices, in Fig. 9 we show the 3D label map obtained by each method. Again, this confirms that the HRMRA dataset is easier to segment, and that, even with a 30% supervision, a GAN-based approach is preferable to the fully supervised one. The difference between 1B and 1D for the MMWHS dataset appears to be visually significant, with very scarce labels and poor boundaries drawn by a UNet and a much better result in case D.

Finally, Table 1 summarizes the average signed differences and root-mean-squared error (RMSE) between HRMRA volumes obtained by the ground truth label maps and the ones calculated from outputs of the trained models. This shows that the calculated volumes are substantially closer to the ground truth values in semi-supervised experiments, rather than in unsupervised ones. The corresponding table for MMWHS data is found in Additional file 1: Table S3.

**Table 1:** Table containing metrics for volume measurements (mL) obtained from label maps

Method	SUP	LV	LVM	RV	LA	RA	AO
GT		99.64 ± 33.14	115.51 ± 26.63	117.32 ± 31.92	57.45 ± 20.31	70.18 ± 21.97	35.90 ± 9.23
VAE	0%	35.6 ± 17.7 (40.5)	37.9 ± 28.6 (47.3)	63.3 ± 25.9 (68.2)	32.2 ± 15.5 (35.7)	26.9 ± 12.4 (29.6)	13.7 ± 9.1 (16.4)
	10%	12.6 ± 10.6 (16.4)	31.4 ± 16.1 (35.2)	39.7 ± 19.9 (44.3)	20.2 ± 16.4 (25.9)	19.4 ± 10.7 (22.1)	8.6 ± 9.8 (13.0)
	20%	6.3 ± 9.2 (11.1)	28.2 ± 13.1 (31.1)	33.8 ± 20.0 (39.2)	13.9 ± 14.5 (20.0)	18.3 ± 9.5 (20.6)	9.3 ± 5.4 (10.7)
	30%	3.4 ± 8.6 (**9.2**)	26.5 ± 11.5 (**28.8**)	24.5 ± 15.7 (**29.0)**	12.2 ± 12.9 **(17.7**)	16.3 ± 11.3 (**19.8**)	7.5 ± 5.4 (**9.2**)
GAN	0%	12.7 ± 12.4 (17.6)	3.9 ± 14.1 (14.5)	20.8 ± 13.1 (24.5)	2.4 ± 16.3 (16.2)	− 0.8 ± 9.2 (***9.2***)	1.8 ± 4.5 (***4.8***)
	10%	2.3 ± 7.2 (7.5)	2.1 ± 8.8 (***8.9***)	7.4 ± 11.2 (13.4)	4.7 ± 13.7 (14.3)	1.2 ± 10.4 (10.3)	1.0 ± 5.2 (5.2)
	20%	0.0 ± 7.6 (***7.5***)	− 1.4 ± 12.4 (12.3)	3.6 ± 11.2 (11.6)	2.0 ± 13.8 (13.8)	− 0.4 ± 9.7 (9.6)	0.6 ± 5.4 (5.4)
	30%	− 1.8 ± 8.1 (8.3)	0.6 ± 9.5 (9.4)	2.2 ± 10.3 (***10.5***)	1.9 ± 10.9 (***11.0***)	0.6 ± 9.6 (9.6)	0.0 ± 5.4 (5.4)
UNet	10%	47.2 ± 75.1 (87.9)	− 5.1 ± 27.4 (27.6)	− 9.8 ± 25.6 (27.1)	7.6 ± 42.5 (42.7)	7.4 ± 32.9 (33.3)	− 5.9 ± 9.3 (10.9)
	20%	97.2 ± 73.2 (121.2)	0.3 ± 14.9 (**14.7**)	− 9.4 ± 18.1 (20.3)	− 1.7 ± 19.7 (**19.5**)	0.4 ± 17.3 (17.1)	1.1 ± 11.1 (11.1)
	30%	15.6 ± 46.6 (**48.7**)	6.7 ± 19.9 (20.8)	− 2.0 ± 18.5 (**18.4**)	3.1 ± 27.2 (27.0)	− 2.6 ± 15.5 **(15.5**)	− 0.3 ± 6.4 (**6.4**)

The results are reported using avg ± std signed differences (RMSE) between ground truth volumes (top row) and predicted volumes. The second column (SUP) refers to the supervision level adopted in the experiment. The best result per each method is highlighted in bold, and the best result overall is bolditalics. HRMRA Dataset

## Discussion

Our results show that adding a small number of supervised cases to a generative modelling domain adaptation method can significantly boost segmentation quality. In our experiments, using two CMRA datasets with different resolution quality, as little as 10% supervision was enough for a significant change from a completely unsupervised approach (Figs. 3, 4). In a situation where very little ground truth is available in one domain, we show that supervised techniques, although optimally trained, are outperformed by generative domain adaptation methods. In the MMWHS dataset, we achieved Dice scores of 0.86 (LV), 0.69 (LVM), 0.74 (RV), 0.73 (LA), 0.79 (RA), 0.74 (AO). This is on average a 12% increase on the original GAN-based approach [8], which reported 0.79 (LV), 0.47 (LVM), 0.62 (LA), 0.65 (AO), and a 10% increase on the results from [20]: 0.74 (LV), 0.47 (LVM), 0.73 (RV), 0.63 (LA), 0.71 (RA) in the original VAE approach. Moreover, in the HRMRA dataset, providing higher resolution in the CMRA domain, we obtained an average Dice of 0.86 across all labels. This compares well with the results of the 2017 Multi-Modality Whole Heart Segmentation (MMWHS) challenge [25], which presented more than 10 algorithms for supervised CTA and CMRA segmentation, with the highest results reporting a maximum 3D Dice of 0.908 $\pm$ 0.086 for CT and 0.874 $\pm$ 0.039 for MR. Compared with interobserver errors from a multi-core-lab study [19], the mean and standard deviation of volume differences between core labs was typically 20 ± 10 mL respectively, compared with − 1.8 ± 8.1 mL + for LV and 2.2 ± 10.3 ml for RV volume (Table 1, 30% supervised GAN). Scan-rescan coefficient of variation for LV mass was 5%, or 7.5 g, in [3] compared with 9.5 g for the 30% supervised GAN (Table 1). Therefore, although our results need to be improved to achieve super-human performance, current results are clinically applicable.

In our previous work [14], the training of the generative modelling techniques was limited by a manual registration step, whereas here we focus on boosting the results by relying solely on the original images and the architecture. Important factors that improved these models included: scaling of input images to the GAN architectures in a specific range and training on random slices rather than consecutive ones extracted from a volume, cropping images around the heart for the VAE approach. The GAN approach, once stably trained, gave generally better results, and can generate fake images from both source and target domains (Figs. 5, 6). This can be very useful in applications where a certain anatomy is required in a specific modality, or, in the future, for a fusion between imaging modalities [18]. On the other hand, the VAE approach was easier to train and twice as fast (~ 12 h for single fold training vs ~ 24 h for GAN-based method) and can still output high-quality segmentations. Although we solely optimise and validate our methods based on segmentation quality, we believe the difference in reconstruction quality to be a significant factor. VAE based methods tend to produce smoother images than GANs, since they are maximum likelihood estimations rather than a Nash equilibrium. Moreover, VARDA’s architecture specifically aims at learning features that are domain-invariant during the training stage, hence the reconstructed images are intended to lose most of the features which would make them look realistic (cf. Figure 6 in [20]).

In addition to Fig. 9, in Additional files 2 and 3 we provide videos which show the 3D consistency of the label maps obtained on this dataset by both generative techniques. Indeed, the results achieved and the quality of the HRMRA data suggest that one could move further by performing an accurate segmentation of the smaller structures (arteries, veins) and thus, generating another interesting application directed at patients in need of cardiac catheterisation [26]. Both the GAN- and VAE- based approach could also be modified to directly work on 3D inputs and tackle 4D challenges such as the segmentation of 3D cardiac CINE MRI, investigating motion and geometry of the heart.

## Limitations

Although in this paper we overcome the previous requirement of multi-modality registration [14], the experiments presented here heavily rely on pre-processing steps which include cropping and centering of the input images. These are challenging to identify and widely vary depending on the technique used. They also appear to significantly affect segmentation performance. Indeed, the cropping step seems to be an essential factor for both networks, and particularly in the VAE-based approach, which requires a very close field of view. In our experiments, this was obtained by identifying the centroid of the heart using the ground-truth labels but in clinical practice the performance would highly benefit from a pre-processing bounding box location step. The generalizability of deep learning methods for segmentation would benefit from increased dataset size and variability in terms of MR scanner, sequence and presence of pathologies. Lastly, we limited evaluation to 6 labels, however, extension to pulmonary veins and arteries is possible in future work since the labels are available in the ground truth dataset.

## Conclusion

In this study, we compared semi-supervised methods based on generative modelling with a state-of-the-art fully supervised one for the task of CMRA segmentation across two datasets with different original resolution. We demonstrated that, in absence of many ground truth cases, a domain adaptation approach is beneficial, and this can be used to accurately segment bigger structures as well as minor ones, and to generate synthetic images of specific imaging modalities.

### Supplementary Information

**Additional file 1: Table S1. **Table of comparison between all methods in Dataset 1 (MMWHS). The percentage of supervision is specified in brackets. Each entry represents Dice (odd rows) and ASD (even rows) average results across entire dataset. Best results are highlighted in red (Dice), and green (ASD). **Table S2.** Table of comparison between all methods in Dataset 2 (HRMRA). The percentage of supervision is specified in brackets. Each entry represents Dice (odd rows) and ASD (even rows) average results across entire dataset. Best results are highlighted in red (Dice), and green (ASD). **Table S3.** Table containing metrics for volume measurements (mL) obtained from label maps. The results are reported using avg ± std signed differences (RMSE) between ground truth volumes (top row) and predicted volumes. The second column refers to the supervision level adopted in the experiment. The best result per each method is highlighted in bold, and the best result overall is color-coded. MMWHS Dataset. **Figure S1.** Results grouped by label. In each boxplot, statistical analysis is conducted between experiments obtained by different methods, as per legend on the top left corner. Dashed brackets for p <= 5.00e−02, square brackets for p <= 1.00e−03. HRMRA Dataset.  **Figure S2.** Results grouped by label. In each boxplot, statistical analysis is conducted between experiments obtained by different methods, as per legend on the top left corner. Dashed brackets for p <= 5.00e-02, square brackets for p <= 1.00e-03. MMWHS Dataset.

**Additional file 2. Video S1. **Show complete volume label maps obtained using the GAN-based and VAE-based approaches respectively for inference on one case from the HRCMRA dataset. Legend for segmentation labels as follows: LV = turquoise, LVM = orange, RV = blue, LA = green, RA = yellow, AO = grey.

**Additional file 3: Video S2. **Show complete volume label maps obtained using the GAN-based and VAE-based approaches respectively for inference on one case from the HRCMRA dataset. Legend for segmentation labels as follows: LV = turquoise, LVM = orange, RV = blue, LA = green, RA = yellow, AO = grey.

## Data Availability

The results and code used during this study is available from the corresponding author on reasonable request.

## Abbreviations

CMR: Cardiovascular magnetic resonance
DL: Deep learning
GAN: Generative adversarial network
VAE: Variational auto encoder
