# Supplementary material for: Combining generative modelling and semi-supervised domain adaptation for whole heart cardiovascular magnetic resonance angiography segmentation
Source: J Cardiovasc Magn Reson. 2023 Dec 20;25:80. doi: 10.1186/s12968-023-00981-6 (PMC10734115; doi:10.1186/s12968-023-00981-6)

**Additional file**

Video S1 and S2 show complete volume label maps obtained using the GAN-based and VAE-based approaches respectively for inference on one case from the HRCMRA dataset.

Legend for segmentation labels as follows: LV = turquoise, LVM = orange, RV = blue, LA = green, RA = yellow, AO = grey.

Table S1: Table of comparison between all methods in Dataset 1 (MMWHS). The percentage of supervision is specified in brackets. Each entry represents Dice (odd rows) and ASD (even rows) average results across entire dataset. Best results are highlighted in red (Dice), and green (ASD).

| **METHOD** | **LV** | **LVM** | **RV** | **LA** | **RA** | **AO** |
| --- | --- | --- | --- | --- | --- | --- |
| VAE-based  (no supervision) | 0.81±0.10 | 0.52±0.12 | 0.58±0.19 | 0.33±0.27 | 0.61±0.17 | 0.49±0.15 |
|  | 6.77±4.51 | 9.88±6.76 | 12.51±8.58 | 22.80±13.19 | 12.24±6.62 | 15.98±6.91 |
| VAE-based  (10% supervision) | 0.83±0.10 | 0.58±0.12 | 0.63±0.18 | 0.55±0.21 | 0.66±0.13 | 0.59±0.13 |
|  | 5.55±4.90 | 8.85±6.21 | 11.76±8.40 | 12.03±6.92 | 11.76±7.35 | 10.36±3.70 |
| VAE-based  (20% supervision) | 0.85±0.08 | 0.64±0.11 | 0.64±0.22 | 0.54±0.29 | 0.71±0.16 | 0.59±0.15 |
|  | **4.76±3.65** | 6.42±4.30 | 11.26±10.25 | 15.22±12.38 | 9.21±8.21 | 11.38±5.44 |
| VAE-based  (30% supervision) | **0.87±0.07** | 0.66±0.10 | 0.68±0.16 | 0.66±0.23 | 0.74±0.11 | 0.63±0.15 |
|  | 4.81±4.50 | 7.03±6.15 | 9.23±6.67 | 7.34±7.69 | 6.66±3.85 | 10.69±4.83 |
| GAN-based  (no supervision) | 0.72±0.13 | 0.53±0.15 | 0.52±0.14 | 0.52±0.33 | 0.71±0.10 | 0.60±0.14 |
|  | 8.87±6.27 | 7.64±5.55 | 11.52±5.55 | 19.57±25.44 | 7.66±3.05 | 9.58±3.93 |
| GAN-based  (10% supervision) | 0.81±0.10 | 0.60±0.13 | 0.64±0.16 | 0.60±0.25 | 0.75±0.10 | 0.59±0.20 |
|  | 7.40±6.04 | 6.24±4.22 | 9.25±5.56 | 9.38±16.36 | 6.56±3.47 | 8.38±4.27 |
| GAN-based  (20% supervision) | 0.84±0.08 | 0.67±0.13 | 0.72±0.12 | 0.73±0.17 | 0.79±0.09 | 0.71±0.13 |
|  | 5.49±4.62 | 4.33±3.91 | 7.12±4.21 | **5.02±3.01** | 5.89±3.76 | 5.88±3.24 |
| GAN-based  (30% supervision) | 0.85±0.08 | **0.69±0.11** | **0.74±0.14** | **0.73±0.20** | **0.79±0.10** | **0.74±0.11** |
|  | 5.93±5.33 | **4.18±4.19** | **7.02±5.78** | 5.18±5.34 | **5.10±3.66** | **5.19±2.99** |
| UNet  (10% supervision) | 0.20±0.19 | 0.25±0.24 | 0.32±0.24 | 0.17±0.17 | 0.34±0.30 | 0.38±0.28 |
|  | 45.86±22.17 | 43.63±25.05 | 24.20±15.54 | 39.05±19.83 | 40.11±30.53 | 25.57±23.12 |
| UNet  (20% supervision) | 0.34±0.22 | 0.40±0.24 | 0.38±0.25 | 0.27±0.26 | 0.44±0.29 | 0.47±0.28 |
|  | 41.72±13.04 | 25.54±23.98 | 20.81±15.38 | 41.99±29.10 | 30.20±32.14 | 20.90±27.05 |
| UNet  (30% supervision) | 0.47±0.20 | 0.60±0.14 | 0.51±0.24 | 0.34±0.24 | 0.62±0.25 | 0.53±0.27 |
|  | 39.90±19.95 | 9.22±7.79 | 21.08±11.74 | 37.16±19.96 | 16.56±26.98 | 8.32±8.34 |

Table S2: Table of comparison between all methods in Dataset 2 (HRMRA). The percentage of supervision is specified in brackets. Each entry represents Dice (odd rows) and ASD (even rows) average results across entire dataset. Best results are highlighted in red (Dice), and green (ASD).

| **METHOD** | **LV** | **LVM** | **RV** | **LA** | **RA** | **AO** |
| --- | --- | --- | --- | --- | --- | --- |
| VAE-based  (no supervision) | 0.81±0.08 | 0.61±0.08 | 0.74±0.06 | 0.67±0.12 | 0.76±0.07 | 0.78±0.07 |
|  | 5.63±3.04 | 6.66±2.77 | 8.78±2.90 | 9.53±3.58 | 7.82±3.47 | 5.13±3.30 |
| VAE-based  (10% supervision) | 0.88±0.05 | 0.74±0.06 | 0.81±0.06 | 0.75±0.11 | 0.80±0.07 | 0.81±0.07 |
|  | 3.20±1.70 | 3.97±1.54 | 5.45±2.31 | 5.76±2.63 | 5.12±2.63 | 3.47±0.98 |
| VAE-based  (20% supervision) | 0.90±0.04 | 0.77±0.05 | 0.83±0.07 | 0.78±0.10 | 0.82±0.06 | 0.82±0.05 |
|  | 2.61±1.28 | 3.55±1.41 | 5.53±4.68 | 4.78±2.14 | 4.78±2.39 | 3.25±1.12 |
| VAE-based  (30% supervision) | 0.90±0.04 | 0.78±0.04 | 0.86±0.05 | 0.80±0.08 | 0.82±0.07 | 0.84±0.05 |
|  | 2.54±1.19 | 3.20±1.21 | 3.72±2.53 | 4.21±2.01 | 4.51±2.89 | 2.78±0.90 |
| GAN-based  (no supervision) | 0.87±0.06 | 0.74±0.07 | 0.83±0.04 | 0.79±0.08 | 0.85±0.05 | 0.83±0.05 |
|  | 3.96±2.44 | 4.05±1.54 | 5.25±1.49 | 4.97±1.62 | 3.72±1.52 | 3.54±1.12 |
| GAN-based  (10% supervision) | **0.91±0.04** | 0.83±0.04 | 0.88±0.05 | 0.81±0.06 | 0.87±0.05 | **0.85±0.04** |
|  | 2.69±1.12 | 2.51±0.72 | 3.71±2.26 | 4.62±1.62 | 3.48±1.75 | 3.15±0.92 |
| GAN-based  (20% supervision) | 0.91±0.04 | 0.82±0.05 | 0.89±0.05 | 0.81±0.07 | 0.86±0.05 | 0.85±0.04 |
|  | 2.27±0.71 | 2.63±1.02 | **3.18±2.54** | 4.34±1.47 | **3.31±1.30** | 2.82±0.80 |
| GAN-based  (30% supervision) | 0.91±0.04 | **0.83±0.05** | **0.89±0.04** | **0.83±0.06** | **0.87±0.05** | 0.85±0.05 |
|  | **2.26±0.76** | **2.39±0.73** | 3.23±1.77 | **3.92±1.30** | 3.33±1.51 | **2.80±0.97** |
| UNet  (10% supervision) | 0.64±0.21 | 0.70±0.08 | 0.69±0.22 | 0.55±0.26 | 0.62±0.21 | 0.72±0.15 |
|  | 28.44±20.38 | 10.14±9.59 | 12.21±10.89 | 22.98±24.90 | 19.59±16.01 | 6.64±4.20 |
| UNet  (20% supervision) | 0.60±0.18 | 0.76±0.07 | 0.81±0.12 | 0.65±0.23 | 0.75±0.16 | 0.76±0.09 |
|  | 37.32±17.25 | 6.13±4.81 | 6.01±4.67 | 17.64±23.19 | 10.80±9.07 | 7.46±7.50 |
| UNet  (30% supervision) | 0.82±0.12 | 0.77±0.06 | 0.84±0.10 | 0.63±0.27 | 0.79±0.09 | 0.80±0.08 |
|  | 13.06±18.36 | 7.88±8.55 | 5.17±3.86 | 19.57±30.24 | 6.87±3.79 | 4.90±2.56 |

Table S3: Table containing metrics for volume measurements (mL) obtained from label maps. The results are reported using avg ± std signed differences (RMSE) between ground truth volumes (top row) and predicted volumes. The second column refers to the supervision level adopted in the experiment. The best result per each method is highlighted in bold, and the best result overall is color-coded. MMWHS Dataset.

| **METHOD** | **SUP.** | **LV** | **LVM** | **RV** | **LA** | **RA** | **AO** |
| --- | --- | --- | --- | --- | --- | --- | --- |
| GT |  | 133.74±48.62 | 134.51±34.82 | 161.93±99.60 | 84.41±51.75 | 135.80±90.78 | 79.50±35.52 |
| VAE | 0% | 14.8±30.6 (33.3) | 58.3±50.1  (76.1) | 63.0±61.7  (87.1) | 31.9±56.8 (63.9) | 22.0±52.1 (55.3) | 106.6±80.6 (132.4) |
|  | 10% | 19.4±39.1 (42.7) | 66.7±60.9  (89.2) | 69.8±77.7  (103.0) | 17.3±45.2  (**47.3**) | 24.2±48.0 (**52.6**) | 54.5±39.9 (66.9) |
|  | 20% | 6.0±18.2  (**18.7**) | 39.0±50.0  (**62.4**) | 56.1±62.2  (82.6) | 27.9±49.8 (56.0) | 19.1±53.7 (55.7) | 67.0±52.2 (84.1) |
|  | 30% | 8.6±19.6 (21.0) | 48.1±52.7  (70.3) | 51.3±64.3  (**81.0**) | 3.0±50.9  (49.7) | 4.4±55.4  (54.2) | 52.7±38.8 (**64.9**) |
| GAN | 0% | -7.5±36.1 (36.0) | 46.8±56.9  (72.6) | 4.0±92.8  (90.5) | 7.6±33.1  (33.1) | -1.2±45.9 (44.7) | 20.6±42.9 (46.6) |
|  | 10% | 15.6±29.2 (32.5) | 12.8±49.3  (49.7) | -1.5±71.8  (70.0) | -19.3±30.7 (35.6) | -13.3±53.1 (53.4) | 1.6±27.0  (26.4) |
|  | 20% | 2.7±16.2 (**16.0**) | 2.4±29.2  (**28.5**) | 0.9±62.3  (**60.7**) | -3.3±20.1 (**19.8**) | -2.7±34.9 (**34.1**) | -2.3±32.1 (31.4) |
|  | 30% | 12.2±22.1 (24.7) | -4.4±38.3  (37.6) | -1.4±67.4  (65.7) | -6.2±23.8 (24.0) | -7.2±43.7 (43.2) | -1.9±24.6 (**24.1**) |
| UNet | 10% | 88.5±191.9 (207.0) | 178.1±400.2 (428.8) | 77.2±91.5  (118.0) | 56.6±157.1 (163.2) | 44.5±141.2 (144.7) | 63.9±193.8 (199.4) |
|  | 20% | 169.8±187.0 (249.1) | -14.3±81.7  (80.9) | -49.3±96.2 (105.9) | -1.6±74.8 (**72.9**) | -34.4±131.3 (132.5) | -25.8±41.7 (**48.1**) |
|  | 30% | 67.0±109.1 (**125.7**) | -21.7±36.0  (**41.2**) | 21.0±99.6  (**99.3**) | 84.6±195.5 (208.5) | -37.1±81.8 (**87.9**) | -35.1±36.6 (50.1) |

Figure S1: Results grouped by label. In each boxplot, statistical analysis is conducted between experiments obtained by different methods, as per legend on the top left corner. Dashed brackets for p <= 5.00e-02, square brackets for p <= 1.00e-03. HRMRA Dataset.


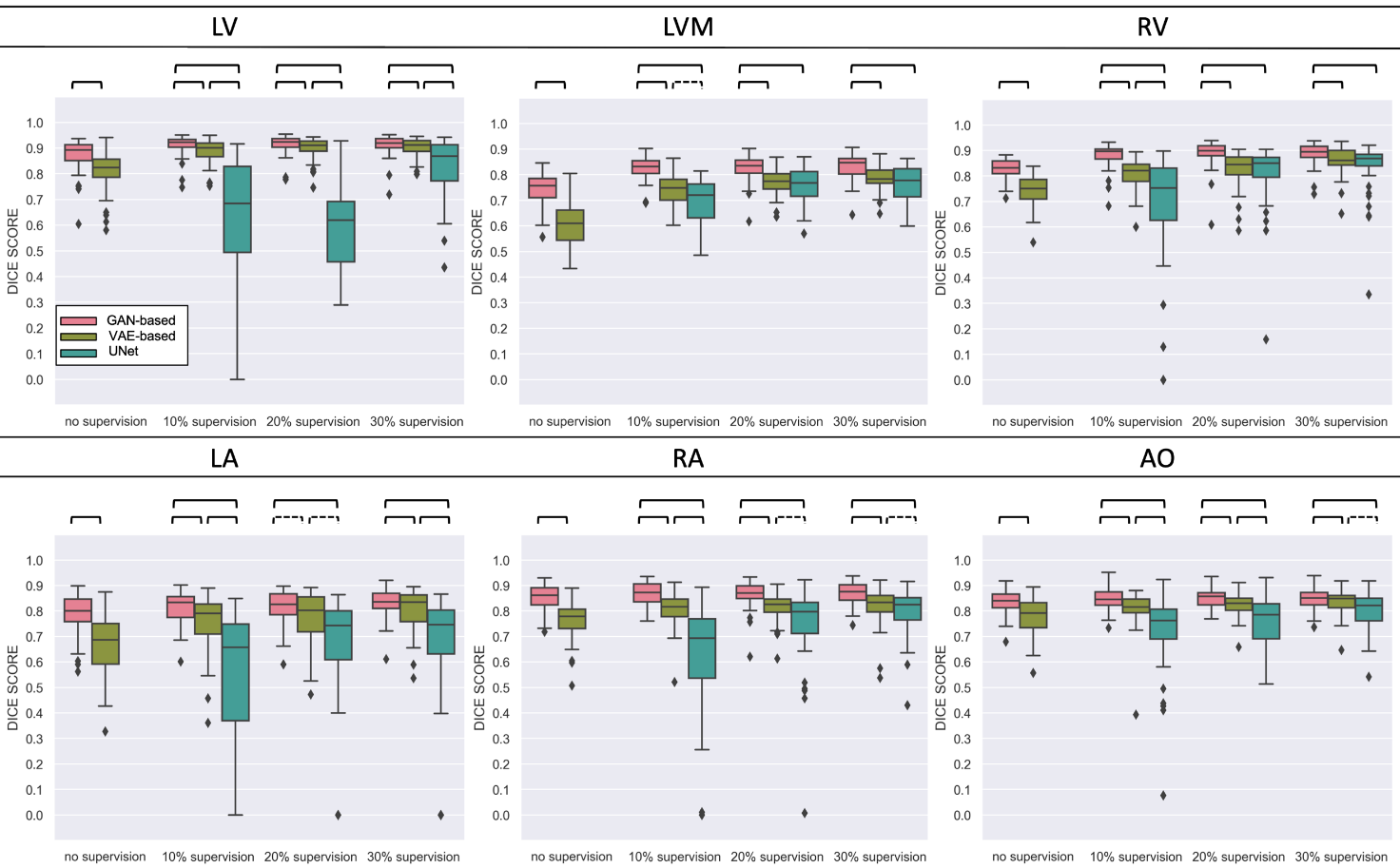


Figure S2: Results grouped by label. In each boxplot, statistical analysis is conducted between experiments obtained by different methods, as per legend on the top left corner. Dashed brackets for p <= 5.00e-02, square brackets for p <= 1.00e-03. MMWHS Dataset.


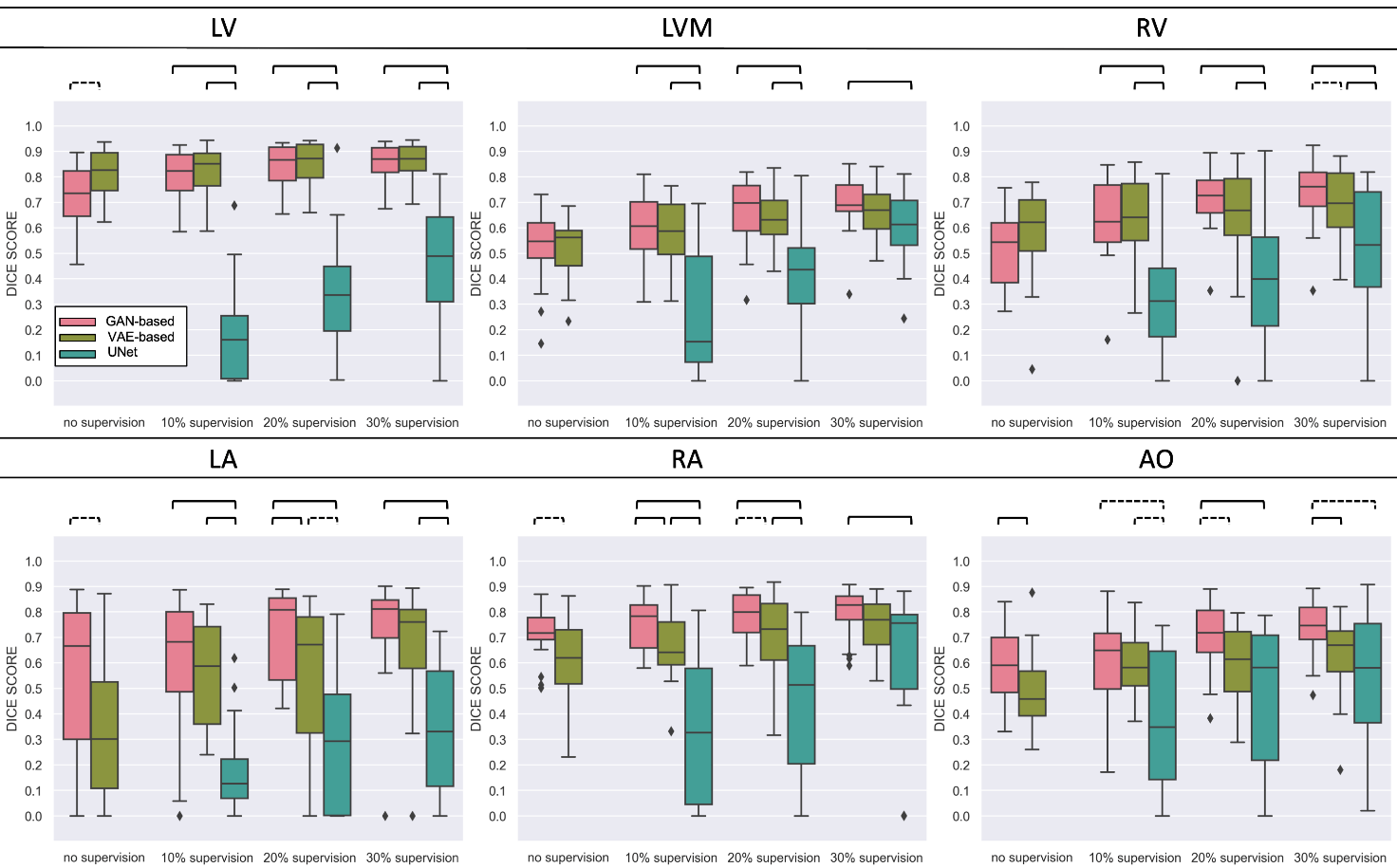

Supplement: Supplementary file 1 — Additional file 1: Table S1. Table of comparison between all methods in Dataset 1 (MMWHS). The percentage of supervision is specified in brackets. Each entry represents Dice (odd rows) and ASD (even rows) average results across entire dataset. Best results are highlighted in red (Dice), and green (ASD). Table S2. Table of comparison between all methods in Dataset 2 (HRMRA). The percentage of supervision is specified in brackets. Each entry represents Dice (odd rows) and ASD (even rows) average results across entire dataset. Best results are highlighted in red (Dice), and green (ASD). Table S3. Table containing metrics for volume measurements (mL) obtained from label maps. The results are reported using avg ± std signed differences (RMSE) between ground truth volumes (top row) and predicted volumes. The second column refers to the supervision level adopted in the experiment. The best result per each method is highlighted in bold, and the best result overall is color-coded. MMWHS Dataset. Figure S1. Results grouped by label. In each boxplot, statistical analysis is conducted between experiments obtained by different methods, as per legend on the top left corner. Dashed brackets for p <= 5.00e−02, square brackets for p <= 1.00e−03. HRMRA Dataset. Figure S2. Results grouped by label. In each boxplot, statistical analysis is conducted between experiments obtained by different methods, as per legend on the top left corner. Dashed brackets for p <= 5.00e-02, square brackets for p <= 1.00e-03. MMWHS Dataset. [file 12968_2023_981_MOESM1_ESM.docx]
